# Relationships between electrolyte and amino acid compositions in sweat during exercise suggest a role for amino acids and K^+^ in reabsorption of Na^+^ and Cl^-^ from sweat

**DOI:** 10.1371/journal.pone.0223381

**Published:** 2019-10-03

**Authors:** Grace R. Murphy, R. Hugh Dunstan, Margaret M. Macdonald, Nattai Borges, Zoe Radford, Diane L. Sparkes, Benjamin J. Dascombe, Timothy K. Roberts

**Affiliations:** University of Newcastle, Callaghan, NSW, Australia; University of Pisa, ITALY

## Abstract

Concentrations of free amino acids and [K^+^] in human sweat can be many times higher than in plasma. Conversely, [Na^+^] and [Cl^-^] in sweat are hypotonic to plasma. It was hypothesised that the amino acids and K^+^ were directly or indirectly associated with the resorption of Na^+^ and Cl^-^ in the sweat duct. The implication would be that, as resources of these components became limiting during prolonged exercise then the capacity to resorb [Na^+^] and [Cl^-^] would diminish, resulting in progressively higher levels in sweat. If this were the case, then [Na^+^] and [Cl^-^] in sweat would have inverse relationships with [K^+^] and the amino acids during exercise. Forearm sweat was collected from 11 recreational athletes at regular intervals during a prolonged period of cycling exercise after 15, 25, 35, 45, 55 and 65 minutes. The subjects also provided passive sweat samples via 15 minutes of thermal stimulation. The sweat samples were analysed for concentrations of amino acids, Na^+^, Cl^-^, K^+^, Mg^2+^ and Ca^2+^. The exercise sweat had a total amino acid concentration of 6.4 ± 1.2mM after 15 minutes which was lower than the passive sweat concentration at 11.6 ± 0.8mM (*p*<0.05) and showed an altered array of electrolytes, indicating that exercise stimulated a change in sweat composition. During the exercise period, [Na^+^] in sweat increased from 23.3 ± 3.0mM to 34.6 ± 2.4mM (*p*<0.01) over 65 minutes whilst the total concentrations of amino acids in sweat decreased from 6.4 ± 1.2mM to 3.6 ± 0.5mM. [Na^+^] showed significant negative correlations with the concentrations of total amino acids (*r* = -0.97, *p*<0.05), K^+^ (*r* = -0.93, *p*<0.05) and Ca^2+^ (*r* = -0.83, *p*<0.05) in sweat. The results supported the hypothesis that amino acids and K^+^, as well as Ca^2+^, were associated with resorption of Na^+^ and Cl^-^.

## Introduction

It has been well documented that eccrine sweat produced for evaporative cooling in humans contains water, electrolytes, lactate, ammonia and urea [1–3]. The electrolytes predominantly consist of Na^+^ and Cl^-^ and to a lesser extent, K^+^, Mg^+^ and Ca^+^. Sweat also contains an array of free amino acids including serine, histidine, ornithine, alanine, glycine and lysine that are present at concentrations several times higher than their corresponding levels in plasma [4–6]. Little is known about the function of free amino acids in sweat or why they do not mirror the relative plasma compositions. It has been proposed that at least some of the relative increases of certain amino acids such as serine, histidine and glutamic acid relates to their function as humectants in the natural moisturising factor (NMF) of the outer layers of the skin [5, 7, 8]. However, small contributions from the NMF would be unlikely to account for the >ten-fold increases in concentration observed in sweat relative to plasma[7].

Estimates of the number of eccrine sweat glands on the human body range between 1.6–4 x 10^6^, with the highest densities located on the palms, finger print ridges and the soles of the feet [9]. It has previously been thought that the primary source of sweat electrolytes was derived from blood plasma filtrate of serous secretion through epithelia that have low transepithelial resistance [3, 10, 11]. More recent perspectives suggest, however, that sweat is a “constructed fluid” primarily under cholinergic control in humans; the process begins with active pumping of Cl^-^ followed by Na^+^ into the secretory coil to a high osmolality which in turn draws water into the lumen to re-equilibrate [2]. In this way, the preliminary sweat fluid produced by the eccrine secretory coil contains Na^+^ and Cl^-^ levels isotonic to plasma [3, 12]. This preliminary fluid is pumped along the duct where Na^+^ and Cl^-^ are then actively resorbed to varying degrees depending on the body location [3, 13].

Ductal resorption of Na^+^ and Cl^-^ has been attributed to the epithelial sodium channel (ENaC) and cystic fibrosis transmembrane regulator (CFTR) channel, occurring when Na^+^ is pumped through the ENaC and Cl^-^ follows passively. This is made possible as the loss of Na^+^ from the lumen of the duct creates an electrochemical gradient allowing Cl^-^ to enter the cell via the CFTR channels [14] which are ATP-dependent and pH sensitive [15]. Additional Cl^-^ in the lumen is exchanged for HCO_3_^-^ via anion exchangers embedded in the luminal cell membrane [16]. The sweat released from the duct of the eccrine sweat glands contains Na^+^ and Cl^-^ ions at 10–30% of the concentration at which they occur in the plasma [17, 18]. In contrast, the level of K^+^ in the final sweat is usually 2–3 times higher than found in plasma [3]. It has been suggested that secretion of K^+^ into the sweat fluid within the duct may occur as a result of the active resorption of Na^+^ but this is not fully understood [2]. Electrolyte resorption in the duct is important in avoiding exercise-associated hyponatremia (EAH), which occurs when the blood [Na^+^] falls below 135 mmol/L [19]. It commonly occurs in endurance athletes due to a combination of hypotonic fluid intake, reduced or no intake of Na^+^ from food and losses of Na^+^ from sweat and urine [20]. Concentrations below 120 mmol/L can result in fatigue, nausea, acute vomiting, acute epileptic seizures and neurological deficits [21].

Serine, histidine, alanine and ornithine are generally found at high levels in sweat compared with corresponding levels in plasma whilst glutamine and proline have been found at relatively low levels [4–7]. To date, little explanation for the high concentrations of amino acids in sweat has been proposed and there is no understanding of why glutamine and proline are selectively conserved. Shaping a detailed model of the cellular mechanisms that contribute to the high concentrations of amino acids in sweat is integral to determining the impacts of sweat facilitated losses of amino acids. Previous research has indicated that amino acid content in sweat decreased over the course of exercise [7]. An earlier study that collected and analysed sweat samples from the lower back of four athletes during exercise noted that [Na^+^] increased gradually for the first 40 minutes of exercise before plateauing at 26.2 mM (± 19.4 mM) [22]. The physiological impacts of increased Na^+^ losses in sweat with time and diminished levels of amino acids may be linked with, and represent a mechanism for, heat adaptation.

Since the concentrations of certain amino acids and K^+^ have been reported at significantly higher concentrations in sweat than in plasma [6, 23, 24], it was hypothesized that these components could be either directly or indirectly involved with the process of resorption of Na^+^ and Cl^-^ in the sweat duct. Alterations in the sweat concentrations of Mg^2+^ and Ca^2+^ could also potentially contribute to the resorption processes. It was thus proposed that if amino acids, K^+^, Mg^2+^ and Ca^2+^ were involved in the resorption of Na^+^ and Cl^-^, then [Na^+^] and [Cl^-^] would be inversely correlated with concentrations of these sweat components over the duration of the exercise period. The aim of the current study was to investigate potential relationships between [Na^+^] and [Cl^-^] with [K^+^], [Mg^2+^], [Ca^2+^] and the amino acids in sweat collected from recreational athletes over a defined period of cycling exercise. The influence of exercise on sweat composition was also tested by comparisons with sweat collected under passive conditions of thermal stimulation.

## Materials and methods

### Ethics statement

All procedures performed in studies involving human participants were in accordance with the ethical standards of the institutional and/or national research committee and with the 1964 Helsinki declaration and its later amendments or comparable ethical standards.

Eleven recreational athletes were recruited from the University of Newcastle’s Ourimbah and Callaghan campuses to participate in a 65-minute cycling session for sweat collection (seven males (weight 85 ±9 Kg, height 1.8 ± 0.05 m) and four females (weight 65 ± 7, height 1.7 ± 0.05 m), with an age of 28 ± 10y. The group undertook an average of 5.2 hours of exercise per week in the two weeks prior to the study (SD ±2.65 hours, range 2–10.5 hours). This research was approved by the University of Newcastle Human Research Ethics Committee (approval number: H-2014-0086) and the participants provided written informed consent prior to inclusion in the study.

All athletes provided a passive sweat sample from a pre-washed forearm by sitting in a heated tent area at 32°C (±0.4°C) for up to 15 minutes. All participants had their forearms washed with mild detergent and warm water up to the elbow, which was then dried with a clean paper towel. The collected sweat was aspirated into a Monovette^™^ and stored at 4°C until analysed. However, only six of the eleven participants produced an adequate sweat sample during the passive sweat collection.

The participants underwent a YMCA Sub Maximal Cycle Ergometer test to determine maximum predicted workload [25, 26]. A plastic bag was sealed around one arm of each participant just below the elbow to collect sweat. The participants rode on a Monark cycle ergometer at 32°C (±0.4°C) and 42–57% relative humidity at 60% predicted VO_2_ max for a total of 65 minutes. Sweat samples were collected following 15, 25, 35, 45, 55 and 65 minutes of exercise. The participant would dismount the cycle ergometer, the bag was removed, each participant’s arm was re-washed and sealed with a clean bag. The sweat collected in the bag was transferred to a Monovette^™^ transport tube which was then stored at 4°C until analysis.

Amino acids in the sweat samples were extracted and processed with a Phenomenex^®^ EZ:faast^™^ sample kit for analyses by gas chromatography flame ionisation detection (GC-FID) as described previously [7]. A Zebron^®^ 50% phenylpolysiloxane, 50% Methylpolysiloxane column (ZB-50) with dimensions 10 m x 0.25 mm and 0.25 μm film thickness, was used to run all samples. A pulsed splitless method was used with a starting temperature of 150°C which increased at 32°C per minute to a holding temperature of 320°C. The flow rate was 2 mL/ minute and the carrier gas was Helium. The run time was 7.64 minutes in total. All GC-FID operations were controlled through Agilent^®^ ChemStation^TM^ software which had been calibrated for each amino acid with standard concentrations of 5, 10, 20, 30 and 40 nm/100 μL against corresponding FID signal peak area. Norvaline (20 nm/100 μL) was added at the beginning of each sample preparation as the internal standard. The amino acids able to be integrated by this method included L-alanine, L-sarcosine, L-glycine, α-aminobutyric acid, L-valine, ß-aminoisobutyric acid, L-leucine, L-alloisoleucine, L-isoleucine, L-threonine, L-serine, L-proline, L-asparagine, L-thioproline, L-aspartic acid, L-methionine, L-hydroxyproline, L-glutamic acid, L-phenylalanine, α-aminoadipic acid, α-aminopimelic acid, L-glutamine, L-ornithine, glycyl-proline (dipeptide), L-lysine, L-histidine, L-hydroxylysine, L-tyrosine, proline-hydroxyproline (dipeptide), L-tryptophan, L-cystathionine and L-cystine. Total amino acid concentrations were based on summation of the measured individual amino acids.

To measure Na^+^, Mg^2+^, K^+^ and Ca^2+^ concentrations in sweat, samples were diluted 1:100 in 2% NHO_3_ in a Labcon^®^ MetalFree 15 mL tube and mixed thoroughly for 30 seconds. The samples were left to digest for 48–72 hours before 10 μL of an Agilent internal standard mix containing 100 ppm of Li6, Sc, Ge, In, Lu, Rh, Tb and Bi was added to each sample to produce a final concentration of 100 ppb. Each sample was run on an Agilent ICP-MS 7900. To determine Na^+^, Mg^2+^, K^+^ and Ca^2+^ concentrations in each sample, High Purity Standards^™^ ICP-AM-15-1M mix was used to make calibration standards of concentrations 0, 1, 5, 10, 50, 100, 500, 1000, 5000 and 10000 ppb. These were prepared freshly and run immediately prior to each batch of sweat samples to produce a 10 point calibration curve employed upon batch completion by Agilent Technologies MassHunter 4.2 Workstation software to quantify [Na^+^]. [Cl^-^] was determined in the same way with an 8 point calibration curve (0 ppb, 0.606 ppb, 6.06 ppb, 60.6 ppb, 0.606 ppm, 6.06 ppm, 15.15 ppm and 30.3 ppm) prepared from a 10, 000 ppm stock solution of Fluka NaCl in Milli Q water. The internal standard mix was added to both calibration standards making a final concentration of 100 ppb.

Electrolyte and amino acid concentrations across the exercise protocol were analysed using one-way repeated measures ANOVAs. Upon significant time effects, further analysis were performed using Duncan’s multiple range test. Additionally, Pearson’s correlations were performed to assess interrelationships between outcome variables. All statistical analyses were conducted using TIBCO Software Inc. (2017), Statistica (data analysis software system), version 13 and statistical significance was accepted at *p*<0.05.

## Results

The evaluations of electrolytes and amino acids in sweat have been summarised in Table 1 with reference to comparisons with literature values for plasma composition (electrolytes [27] and [28]; plasma [29]). [Na^+^] and [Cl^-^] were present in the passive sweat at 10% of the plasma concentrations, [Mg^2+^] at 22% and [Ca^2+^] at 34%, whereas [K^+^] was two times more concentrated than in plasma. The average total level of amino acids was nearly four times higher in the passive sweat compared with the literature values for plasma. Serine, glycine, alanine, ornithine and aspartic acid were the most abundant amino acid components in the sweat. Aspartic acid was 100 times more concentrated in the passive sweat than plasma and serine, ornithine and glycine were 26-fold, 14-fold and 7.4-fold higher than the corresponding literature plasma levels. Only trace levels of glutamine were detected in the passive sweat (Table 1).

**Table 1:** The average concentrations of the electrolytes and amino acids in sweat collected from the recreational athletes were measured at six time points over the 65-minute period of exercise for comparison with literature plasma concentrations. Passive sweat samples were collected from six participants.

Electrolyte and amino acid concentrations (μM)	*Passive sweat* (n = 6)	Literature Plasma μM ± SE	Exercise sweat (n = 11)						Correlations	Na^+^	Cl^-^	K^+^	Mg^2+^	Ca^2+^
			15 min	25 min	35 min	45 min	55 min	65 min						
Na^+^	14,385 ± 1226	140,400 ± 500	23,275 ± 3,053	28,703 ± 3,146	30,481 ± 2,950	30,488 ± 2,220	32,291 ± 2445	34,598 ± 2,366^	Na^+^		*0*.*99*	*-0*.*93*	-0.65	*-0*.*83*
Cl^-^	10,910 ± 1945	104,500 ± 500	17,163 ± 4,293	22,157 ± 3,922	25,335 ± 3,758	24,840 ± 2,031	25,573 ± 2789	28,595 ± 3,105	Cl^-^			*-0*.*93*	-0.65	*-0*.*83*
K^+^	10,358 ± 685	4,420 ± 80	8,224 ± 929	6,250 ± 600	5,908 ± 639	5,517 ± 631	5,548 ± 625	5,489 ± 503	K^+^				*0*.*82*	*0*.*96*
Mg^2+^	211 ± 26	964 ± 13	131 ± 24*	91 ± 15	93 ± 14	98 ± 14	100 ± 12	104 ± 15	Mg^2+^					*0*.*95*
Ca^2+^	840 ± 95	2,483 ± 14	555 ± 75*	324 ± 43	331 ± 44	313 ± 33	331 ± 33	333 ± 35^						
Serine	3,030 ± 226	114	1,835 ± 336*	1,454 ± 285	1,217 ± 267	1,241 ± 193	1,013 ± 186	1,004 ± 203	Serine	*-0*.*98*	*-0*.*97*	*0*.*94*	0.64	*0*.*82*
Glycine	1,751 ± 150	236	997 ± 202*	804 ± 169	689 ± 154	700 ± 120	580 ± 99	595 ± 87	Glycine	*-0*.*97*	*-0*.*96*	*0*.*94*	0.66	*0*.*83*
Alanine	958 ± 96	419	601 ± 123	475 ± 102	426 ± 100	429 ± 79	342 ± 63	348 ± 53	Alanine	*-0*.*98*	*-0*.*95*	*0*.*93*	0.64	*0*.*82*
Ornithine	928 ± 144	65	452 ± 85*	327 ± 60	272 ± 50	289 ± 43	273 ± 36	291 ± 40	Ornithine	*-0*.*89*	*-0*.*90*	*0*.*97*	*0*.*86*	*0*.*95*
Aspartic acid	702 ± 72	7	415 ± 99	299 ± 61	281 ± 60	277 ± 47	244 ± 41	245 ± 34	Aspartic acid	*-0*.*97*	*-0*.*95*	*0*.*98*	0.79	*0*.*92*
Threonine	661 ± 45	146	412 ± 77	319 ± 61	277 ± 60	283 ± 43	216 ± 34	211 ± 42	Threonine	*-0*.*98*	*-0*.*96*	*0*.*92*	0.62	0.80
Histidine	700 ± 73	89	260 ± 26	190 ± 30	159 ± 23	170 ± 15	134 ± 16	149 ± 19^	Histidine	*-0*.*95*	*-0*.*93*	*0*.*96*	0.75	*0*.*88*
Valine	433 ± 36	252	257 ± 48*	208 ± 42	178 ± 39	187 ± 31	139 ± 29	152 ± 20	Valine	*-0*.*95*	*-0*.*93*	*0*.*90*	0.62	0.78
Glutamic acid	393 ± 47	60	168 ± 31*	140 ± 27	122 ± 21	121 ± 12	95 ± 11	106 ± 14	Glutamic acid	*-0*.*94*	*-0*.*92*	*0*.*91*	0.60	0.78
Leucine	335 ± 35	160	162 ± 18*	118 ± 13	102 ± 13	103 ± 8	95 ± 8	95 ± 8^	Leucine	*-0*.*95*	*-0*.*96*	*0*.*99*	0.80	*0*.*93*
Proline	252 ± 33	239	153 ± 30	114 ± 24	101 ± 22	106 ± 17	87 ± 14	89 ± 13	Proline	*-0*.*97*	*-0*.*96*	*0*.*96*	0.74	*0*.*88*
Tyrosine	292 ± 33	72	130 ± 20*	93 ± 15	78 ± 14	77 ± 9	69 ± 8	69 ± 7^	Tyrosine	*-0*.*96*	*-0*.*96*	*0*.*98*	0.76	*0*.*91*
Isoleucine	224 ± 22	84	129 ± 19*	94 ± 15	81 ± 14	83 ± 11	57 ± 12	67 ± 11^	Isoleucine	*-0*.*95*	*-0*.*92*	*0*.*93*	0.67	*0*.*83*
Lysine	265 ± 38	198	110 ± 20*	82 ± 14	66 ± 13	67 ± 10	53 ± 8	65 ± 8^	Lysine	*-0*.*91*	*-0*.*90*	*0*.*96*	0.74	*0*.*88*
Phenylalanine	221 ± 25	65	95 ± 11*	67 ± 8	56 ± 8	57 ± 5	52 ± 5	53 ± 5^	Phenylalanine	*-0*.*94*	*-0*.*95*	*0*.*99*	*0*.*81*	*0*.*93*
Hydroxy proline	93 ± 81		82 ± 63	62 ± 50	47 ± 40	41 ± 33	8 ± 6	30 ± 29	Hydroxy proline	*-0*.*87*	*-0*.*82*	*0*.*84*	0.49	0.69
Asparagine	144 ± 15	49	79 ± 20*	60 ± 14	47 ± 13	47 ± 13	40 ± 13	43 ± 8	Asparagine	*-0*.*94*	*-0*.*94*	*0*.*96*	0.70	*0*.*86*
Tryptophan	87 ± 15	30	38 ± 7*	24 ± 6	18 ± 6	17 ± 4	13 ± 4	13 ± 3^	Tryptophan	*-0*.*97*	*-0*.*96*	*0*.*98*	0.74	*0*.*90*
Glutamine	4 ± 4	645	17 ± 9	13 ± 9	12 ± 9	5 ± 5	10 ± 6	9 ± 7	Glutamine	-0.73	-0.74	*0*.*83*	0.50	0.72
Methionine	43 ± 7	32	11 ± 3*	6 ± 2	4 ± 3	4 ± 2	4 ± 3	6 ± 2^	Methionine	-0.76	-0.79	*0*.*92*	*0*.*88*	*0*.*92*
Hydroxylysine	8 ± 7		8 ± 6	9 ± 8	5 ± 5	6 ± 6	3 ± 3	2 ± 2	Hydroxylysine	*-0*.*82*	-0.81	0.62	0.16	0.40
α-aminoadipic acid	14 ± 6		6 ± 4	5 ± 4	5 ± 5	2 ± 2	2 ± 2		α-aminoadipic acid	*-0*.*88*	*-0*.*86*	0.78	0.33	0.61
α-aminobutyric acid	5 ± 5		5 ± 4	22 ± 19					α-butyric acid	-0.33	-0.39	0.23	-0.23	-0.03
Total	11,584 ± 831	2,962	6,422 ± 1187*	4,975 ± 951	4,231 ± 870	4,305 ± 632	3,534 ± 541	3,639 ± 497	Total	*-0*.*97*	*-0*.*96*	*0*.*95*	0.69	*0*.*85*

The significant differences between the passive sweat and the sweat collected after 15 minutes of exercise have been marked at *p*<0.05 (*). Significant differences during the exercise period are marked ^ (*p*<0.05). Significant correlations between the mean values of Na^+^, Cl^-^, K^+^, Mg^2+^ and Ca^2+^ and the twenty-three most abundant amino acids over the six exercise time points have been marked in italics (*p*<0.05).

The first sweat samples taken after 15 minutes of exercise showed marked differences in composition to the passive sweat samples (Table 1). The mean [Na^+^] and [Cl^-^] were increased by 8.9 mM and 6.25 mM respectively after 15 minutes of exercise compared with passive sweat, although these changes were not statistically significant. The [K^+^] was reduced by 2.1 mM and there were significant reductions in [Mg^2+^] and [Ca^2+^] of 0.08 and 0.29 mM respectively (*p*<0.05). There was a significant reduction in the average total level of amino acids compared to the passive sweat (6.4 vs 11.6 mM) (*p*<0.05). Individual amino acids serine, glycine, ornithine, valine, glutamic acid, leucine, tyrosine, isoleucine, lysine, phenylalanine, asparagine, tryptophan and methionine were significantly lower in the exercise sweat at 15 minutes than in the passive sweat samples (*p*<0.05, Table 1).

**Fig 1:**
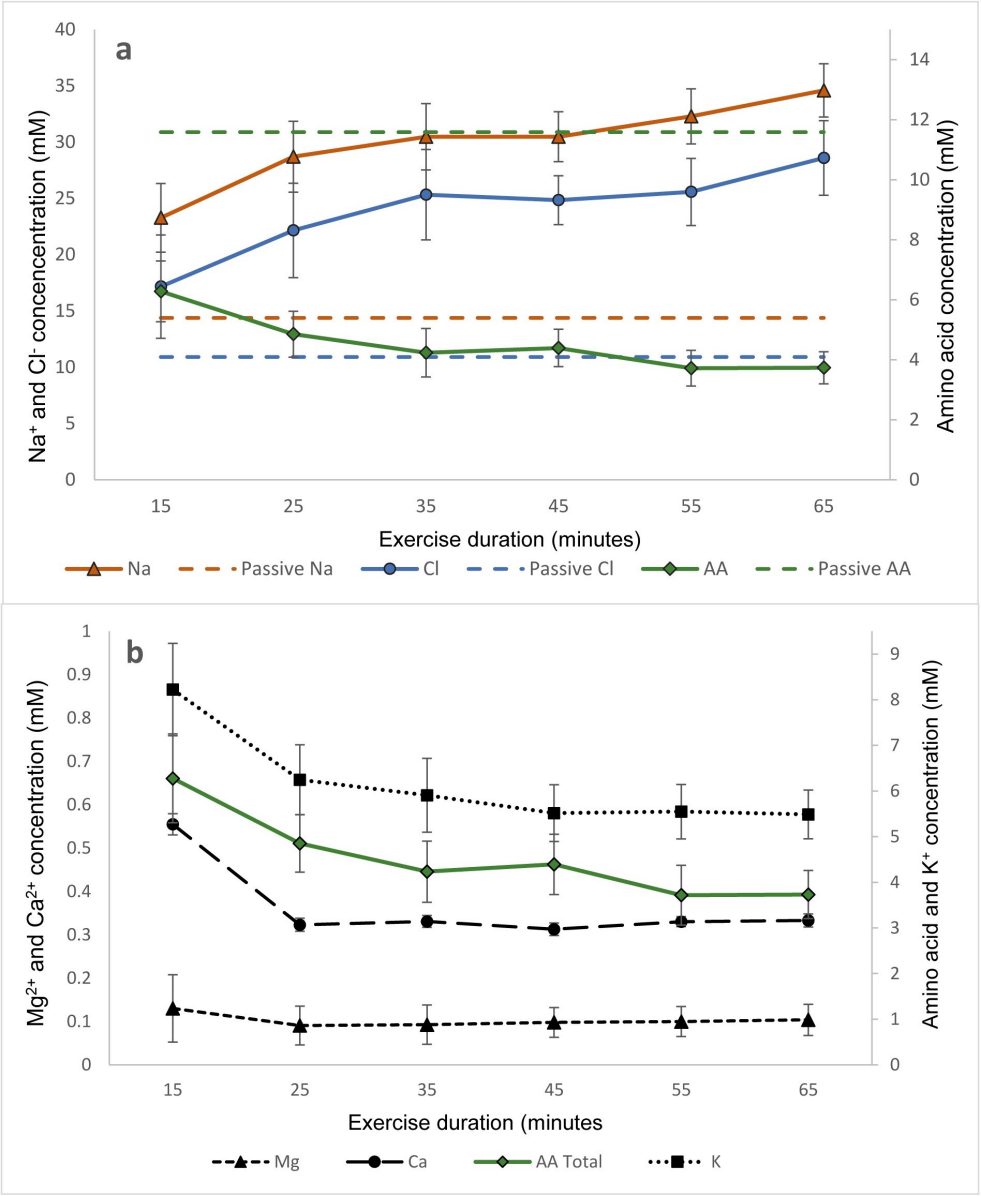
**(a)** Concentrations of Na^+^ and Cl^-^ (primary axis) and mean total amino acid concentrations (secondary axis) in exercise sweat (mM) assessed at ten minute intervals from 15 to 65 minutes of exercise (n = 11). The concentrations of Na^+^, Cl^-^ and total amino acids in passive sweat (n = 6) are also shown for reference on the respective axes. **(b)**, Mg^2+^ and Ca^2+^ concentrations (primary axis) in exercise sweat for the exercise duration are shown with amino acid and K^+^ concentrations (secondary axis).

Electrolyte concentrations changed over the duration of the exercise period. The [Na^+^] and [Cl^-^] increased from 23.3mM and 17.2mM at 15 minutes (*p<*0.05), to 34.6 mM and 28.6 mM respectively at 65 minutes (Fig 1(A)). In contrast, the total amino acid concentrations in sweat reduced from 6.4 mM at 15 minutes to 3.6 mM at 65 minutes although this was not significant. The [K^+^] in exercise sweat declined from 8.2mM to 5.5mM at 65 minutes, but the [Mg^2+^] remained relatively constant throughout the period of exercise (Fig 1(B)). [Ca^2+^] decreased between 15 and 25 minutes but remained stable for the remaining 50 minutes of exercise.

As predicted, the [Na^+^] and [Cl^-^] in the exercise sweat were positively correlated throughout the exercise period (*r* = 0.99, *p*<0.05, Table 1). Neither [Na^+^] nor [Cl^-^] were positively associated with any other electrolyte or amino acid in the sweat but showed significant negative correlations with the total amino acid concentrations (*r* = -0.97, *r* = -0.96, *p*<0.05), K^+^ (*r* = -0.93,*p*<0.05) and Ca^2+^ (*r* = -0.83, *p*<0.05). Significant negative associations were demonstrated between [Na^+^] and twenty of the individual amino acids measured, while [Cl^-^] was negatively correlated with nineteen of the twenty three amino acids assessed during the exercise period (Table 1). The [K^+^], [Mg^2+^], [Ca^2+^] in sweat were all positively correlated with each other (*r* > 0.82, *p*<0.05). [K^+^] was positively correlated with twenty of the amino acids (*p*<0.05), [Ca^2+^] was correlated with fifteen amino acids (*p*<0.05) and [Mg^2+^] was associated with three amino acids (*p*<0.05).

## Discussion

The current study aimed to investigate potential relationships between [Na^+^] and [Cl^-^] with [K^+^], [Mg^2+^], [Ca^2+^] and the amino acids in passive and exercise sweat collected from recreationally active participants over a defined period of cycling exercise. The results indicated that passive sweat collected over a 15 minute period had higher [K^+^], [Mg^2+^] and [Ca^2+^] and concentrations of amino acids compared to sweat collected after 15 minutes of exercise. In contrast, the [Na^+^] and [Cl^-^] in the passive sweat were lower than those observed in the 15 minute exercise sweat. These differential profiles of amino acids and electrolytes in the passive and exercise sweats suggested that the composition of sweat was physiologically regulated by exercise. The advent of exercise would require the body to maintain sweating capacity to counter the higher heat generation for energy consumption in the muscles for prolonged periods and so a more conservative approach for retention of Na^+^ and Cl^-^ would be required. Under these conditions of exercise, the body would limit the rate of losses of the amino acids and K^+^, Ca^2+^ and Mg^2+^ and, as a result, the capacity to resorb the Na^+^ and Cl^-^ would be reduced, resulting in higher [Na^+^] and [Cl^-^] in exercise sweat. It should be noted that K^+^ was the only ion in the exercise sweat at a concentration (8.2mM) greater than the literature average value in plasma (4.4mM). This would be consistent with K^+^ having a major role in the process of sweat formation and/or resorption of Na^+^.

During the exercise period, it was observed that there were progressive increases in in [Na^+^] and [Cl^-^] in the sweat, whilst the [K^+^], [Mg^2+^] and [Ca^2+^] and amino acids diminished. In the current study, the exercise intensity and environmental conditions were kept constant to enable a constant sweat rate [6, 30]. The progressive increases in [Na^+^] and [Cl^-^] were thus interpreted to indicate that the capacity to resorb Na^+^ and Cl^-^ during passage through the duct diminished over the exercise period. In contrast, the [K^+^] in the sweat fell after 15 minutes of exercise and continued to decrease over time to levels that were closer to, but still higher than, the literature plasma levels by 65 minutes. The mean total level of amino acids was also approximately twice the literature plasma value after 15 minutes exercise and progressively diminished over time approaching levels equivalent to plasma by 55–65 minutes. As a result, there were strong positive correlations between the sweat concentrations of amino acids and [K^+^], with both these components having strong negative correlations with [Na^+^] and [Cl^-^]. These results suggested inverse physiological relationships between [Na^+^] and [Cl^-^] in the sweat with [K^+^] and the amino acids which were modulated by the onset and duration of exercise.

The *in vivo* amino acid content of the primary sweat fluid produced in the secretory coil has not been examined independently of sweat leaving the resorptive duct, as was achieved with *in vitro* studies of sweat electrolytes from excised glands [31]. It would be reasonable to assume that the amino acid levels in the primary sweat fluid formed in the secretory coil would be less than or similar to plasma levels with no published literature evidence of amino acid transporters operating in this part of the gland or throughout the transdermal duct. A potential transporter that facilitates the exchange of amino acids for extracellular substrates is the ASCT-1 protein of the alanine/serine/cysteine transporter family (ASCT) [32]. The expression of ASCT-1 mRNA was found to be expressed in all the human tissue types that were tested by northern blot, with the highest abundances occurring in the brain, muscle and pancreatic tissue [33]. The ASCT-1 protein transports alanine, serine, cysteine and threonine in a symmetrical and electroneutral exchange of one of these zwitterionic amino acids with one Na^+^ ion [34]. As the ASCT-1 has a high affinity for serine and alanine, the presence of this transporter in the duct of the sweat gland may provide a supplementary mechanism by which Na^+^ is reclaimed at the cost of small, neutral amino acids. The operation of this transporter would be consistent with the very high levels of serine and alanine measured in the sweat. Other amino acid transport systems are likely to be involved given the strong inverse correlations between [Na^+^] and [Cl^-^] with the concentrations of eighteen amino acids, [K^+^], [Mg^2+^] and [Ca^2+^] in the sweat.

Once the primary sweat fluid has been formed in the secretory coil, the amino acids and K^+^ could be transferred to the primary sweat fluid during passage through the intradermal duct to balance the resorption of Na^+^ and Cl^-^. It has been proposed that K^+^ is lost from the duct cells via leak channels as a consequence of Na^+^ resorption in an attempt to maintain osmotic balance in the cells but not necessarily as a direct part of the Na^+^ transport channel operation [18, 31]. In this context, Na^+^ would be resorbed from the primary sweat fluid into the cells via the ENaC channels as it travels along the duct. The change in the ion concentration gradient causes K^+^ to exit the cell passively through K^+^ leakage channels, resulting in elevated K^+^ concentrations in the sweat at the skin surface. The amino acids may be linked to this process or have independent mechanisms of transfer. Ca^2+^ and Mg^2+^were also implicated in the resorption process by showing positive correlations with K^+^ and the amino acids. Whilst Ca^2+^ has been linked to KCl efflux channels [24, 35, 36], the full role and mode of action of Mg^2+^and Ca^2+^ has not been clearly elucidated and the connection between the cations and amino acids is at present undefined.

The increases in [Na^+^] and [Cl^-^] throughout the exercise period indicated that the capacity for resorption diminished over this period. It was thus proposed that whilst sufficient amino acids and K^+^ were available, the Na^+^ can be effectively resorbed but as exercise and sweating continues and reserves of amino acids and K^+^ diminish, then Na^+^ (and Cl^-^) would not be as effectively resorbed and thus Na^+^ and Cl^-^ would begin to increase in concentration within the sweat matrix. If the concentrations of amino acids and K^+^ became limiting within the duct cells with exercise duration, then fewer Na^+^ ions could be resorbed progressively resulting in higher Na^+^ and lower K^+^ concentrations in sweat as was observed in the present study.

## Conclusions

The data from this study supported the hypothesis that key amino acids in combination with K^+^, Mg^2+^ and Ca^2+^, are transferred into the sweat to facilitate resorption of Na^+^ and Cl^-^. The involvement of the amino acids, K^+^, Mg^2+^ and Ca^2+^ in the resorption of Na^+^ and Cl^-^ would provide an explanation for the high losses of these components via sweat during exertion. During physical activity, reclamation of Na^+^ and Cl^-^ is important in avoiding exercise-associated hyponatremia (EAH). The implication for professional and recreational athletes is that provision of electrolytes alone would not be sufficient to balance the replenishment requirements generated by prolonged exertion. These results could provide new insights in sports medicine to manage athletes undertaking high intensity and endurance training or competition, especially in warmer climates. It would even be possible to use measures of sweat-facilitated losses of amino acids to customise supplementation to minimize the supply from endogenous turnover of body proteins. Ideally, the amino acids and electrolytes would be replaced in a similar concentration profile as to that in which they are lost, if possible during, or immediately after exercise.

## References

[1] Taylor N, Machado-Moreira CA. Regional variations in transepidermal water loss, eccrine sweat gland density, sweat secretion rates and electrolyte composition in resting and exercising humans. Extreme Physiology & Medicine. 2013;2(4). 10.1186/2046-7648-2-4 PMC3710196 23849497

[2] Sonner Z, Wilder E, Heikenfeld J, Kasting G, Beyette F, Swaile D, et al The microfluidics of the eccrine sweat gland, including biomarker partitioning, transport, and biosensing implications. Biomicrofluidics. 2015;9(3):031301 10.1063/1.4921039 26045728 PMC4433483

[3] Sato K. The physiology, pharmacology, and biochemistry of the eccrine sweat gland Reviews of Physiology, Biochemistry and Pharmacology, Volume 79 Berlin, Heidelberg: Springer Berlin Heidelberg; 1977 p. 51–131. 21440 10.1007/BFb0037089

[4] Liappis N, Hungerland H. Quantitative study of free amino acids in human eccrine sweat during normal conditions and exercise. The American Journal of Clinical Nutrition. 1972;25(7):661–3. 10.1093/ajcn/25.7.661 5035052

[5] Kutyshenko VP, Molchanov M, Beskaravayny P, Uversky VN, Timchenko MA. Analyzing and Mapping Sweat Metabolomics by High-Resolution NMR Spectroscopy. PLoS ONE. 2011;6(12):e28824 10.1371/journal.pone.0028824 22194922 PMC3237544

[6] Gitlitz PH, Sunderman, F. William, Hohnadel DC. Ion-Exchange Chromatography of Amino Acids in Sweat Collected from Healthy Subjects during Sauna Bathing. Clinical Chemistry. 1974;20(10):1305–12. 4413985

[7] Dunstan RH, Sparkes DL, Dascombe BJ, Macdonald MM, Evans CA, Stevens CJ, et al Sweat Facilitated Amino Acid Losses in Male Athletes during Exercise at 32–34°C. PLoS One. 2016;11(12):e0167844 10.1371/journal.pone.0167844 27936120 PMC5148013

[8] Rawlings AV, Scott IR, Harding CR, Bowser PA. Stratum corneum moisturization at the molecular level. Journal of investigative dermatology. 1994;103(5):731–40. 10.1111/1523-1747.ep12398620 7963664

[9] Szabo G. The regional anatomy of the human integument with special reference to the distribution of hair follicles, sweat glands and melanocytes. Philos Trans R Soc Lond B Biol Sci. 1967;252(779):447–85.

[10] Fromter E, Diamond J. Route of passive ion permeation in epithelia. Nat New Biol. 1972;235(53):9–13. 10.1038/newbio235009a0 4502409

[11] Lichton I. Osmotic pressure of human sweat. Journal of applied physiology. 1957;11(3):422–4. 10.1152/jappl.1957.11.3.422 13480953

[12] Brusilow S, Gordes E. Determination of sweat gland precursor fluid osmolarity by direct cryoscopy. J clin Invest. 1963;42:920–1.

[13] Patterson MJ, Galloway SD, Nimmo MA. Variations in regional sweat composition in normal human males. Experimental physiology. 2000;85(6):869–75. Epub 2001/02/24. . 11187982 10.1111/j.1469-445x.2000.02058.x

[14] Reddy M, Light M, Quinton P. Activation of the epithelial Na+ channel (ENaC) requires CFTR Cl-channel function. Nature. 1999;402(6759):301–4. 10.1038/46297 10580502

[15] Reddy M, Quinton P. Rapid regulation of electrolyte absorption in sweat duct. The Journal of membrane biology. 1994;140(1):57–67. 10.1007/bf00234486 7519679

[16] Bovell D, Quinton P, editors. The immunocytochemical localisation of anion exchangers (AE) in the reabsorptive duct of the human eccrine sweat gland JOURNAL OF PHYSIOLOGY-LONDON; 2002: CAMBRIDGE UNIV PRESS 40 WEST 20TH ST, NEW YORK, NY 10011–4221 USA.

[17] Schwartz IL, Thaysen JH. Excretion of sodium and potassium in human sweat. J Clin Invest. 1956;35. 10.1172/JCI103245 PMC438784 13278407

[18] Sato K. The mechanism of eccrine sweat secretion. Perspectives in exercise science and sports medicine. Exerc Heat Thermoregul. 1993;6:85–118.

[19] Hew-Butler T, Loi V, Pani A, Rosner MH. Exercise-associated hyponatremia: 2017 update. Frontiers in medicine. 2017;4:21 10.3389/fmed.2017.00021 28316971 PMC5334560

[20] O'Connor RE. Exercise-induced hyponatremia: causes, risks, prevention, and management. Cleveland Clinic journal of medicine. 2006;73(3):S13. 10.3949/ccjm.73.suppl_3.s13 16970148

[21] Nigro N, Winzeler B, Suter‐Widmer I, Schuetz P, Arici B, Bally M, et al Symptoms and characteristics of individuals with profound hyponatremia: a prospective multicenter observational study. Journal of the American Geriatrics Society. 2015;63(3):470–5. 10.1111/jgs.13325 25735607

[22] Schazmann B, Morris D, Slater C, Beirne S, Fay C, Reuveny R, et al A wearable electrochemical sensor for the real-time measurement of sweat sodium concentration. Analytical Methods. 2010;2(4):342–8.

[23] Sato K, Feibleman C, Dobson RL. The electrolyte composition of pharmacologically and thermally stimulated sweat: a comparative study. J Invest Dermatol. 1970;55. 10.1111/1523-1747.ep12260581 5489084

[24] Sato K, Kang WH, Saga K, Sato KT. Biology of sweat glands and their disorders. I. Normal sweat gland function. J Am Acad Dermatol. 1989;20. 10.1016/s0190-9622(89)70081-5 2654213

[25] Golding LA, Myers CR, Sinning WE. Y's way to physical fitness: YMCA of the USA; 1982.

[26] Beekley MD, Brechue WF, Dehoyos DV, Garzarella L, Werber-Zion G, Pollock ML. Cross-validation of the YMCA submaximal cycle ergometer test to predict VO2max. Research quarterly for exercise and sport. 2004;75(3):337–42. 10.1080/02701367.2004.10609165 15487296

[27] Wilkerson J, Horvath S, Gutin B, Molnar S, Diaz F. Plasma electrolyte content and concentration during treadmill exercise in humans. Journal of applied physiology: respiratory, environmental and exercise physiology. 1982;53(6):1529. 10.1152/jappl.1982.53.6.1529 7153149

[28] Walser M. Ion association. VI. Interactions between calcium, magnesium, inorganic phosphate, citrate and protein in normal human plasma. J Clin Invest. 1961;40(4):723–30. 13782899 10.1172/JCI104306 PMC373170

[29] Armstrong MD, Stave U. A study of plasma free amino acid levels. II. Normal values for children and adults. Metabolism. 1973;22(4):561–9. Epub 1973/04/01. 10.1016/0026-0495(73)90069-3 . 4696900

[30] Fukuba Y, Endo MY, Kondo A, Kikugawa Y, Miura K, Kashima H, et al Brachial artery blood flow dynamics during sinusoidal leg cycling exercise in humans. Physiological reports. 2017;5(19). 10.14814/phy2.13456 PMC5641938 28989117

[31] Sato K. Sweat induction from an isolated eccrine sweat gland. American Journal of Physiology-Legacy Content. 1973;225(5):1147–52. 10.1152/ajplegacy.1973.225.5.1147 4745213

[32] Christensen HN, Liang M, Archer EG. A distinct Na+-requiring transport system for alanine, serine, cysteine, and similar amino acids. Journal of Biological Chemistry. 1967;242(22):5237–46. 6070848

[33] Arriza J, Kavanaugh MP, Fairman WA, Wu Y-N, Murdoch GH, North RA, et al Cloning and expression of a human neutral amino acid transporter with structural similarity to the glutamate transporter gene family. Journal of Biological Chemistry. 1993;268(21):15329–32. 8101838

[34] Zerangue N, Kavanaugh MP. ASCT-1 is a neutral amino acid exchanger with chloride channel activity. Journal of Biological Chemistry. 1996;271(45):27991–4. 10.1074/jbc.271.45.27991 8910405

[35] Cui CY, Schlessinger D. Eccrine sweat gland development and sweat secretion. Experimental dermatology. 2015;24(9):644–50. Epub 2015/05/28. 10.1111/exd.12773 26014472 PMC5508982

[36] Cui CY, Sima J, Yin M, Michel M, Kunisada M, Schlessinger D. Identification of potassium and chloride channels in eccrine sweat glands. Journal of dermatological science. 2016;81(2):129–31. Epub 2015/12/03. 10.1016/j.jdermsci.2015.11.001 26627722 PMC5526211

